# Associations Between Physical Fitness, Objectively Measured Physical Activity and Academic Performance

**DOI:** 10.3389/fpubh.2021.778837

**Published:** 2021-12-09

**Authors:** Saša Ðurić, Špela Bogataj, Vinko Zovko, Vedrana Sember

**Affiliations:** ^1^Faculty of Sports, University of Ljubljana, Ljubljana, Slovenia; ^2^Department of Nephrology, University Medical Centre Ljubljana, Ljubljana, Slovenia; ^3^School of Economics and Business, University of Ljubljana, Ljubljana, Slovenia

**Keywords:** physical activity, academic performance, girls, physical fitness, mathematic grade, grade point average

## Abstract

There is evidence that physical activity (PA) can improve the academic performance. We recruited healthy adolescent girls, aged 11–12 years, and measured their PA with the accelerometer ActiGraph GT3X for the consecutive 5 days. Physical fitness (PF) was measured with eight motoric tests and three anthropometry measures. Academic performance (AP) was assessed for the six academic narrated school subjects. The results revealed that the girls were more physically active during the week days and less active at weekend (557 vs. 516 counts/min). Physical education grade shows the highest overall correlations with the results of the PF test battery (*r* = 0.53–0.95, *p* < 0.01). Nevertheless, correlations surprisingly decrease for the combined daily PA (*r* = 0.45), especially the weekend PA (*r* = 0.28). Grade point average and PF correlated moderately (*r* = 0.43-0.64), while they were moderate to high for PA (*r* = 0.59–0.87). Many questions arose after the completion of the present study and several new topics opened up, such as the question of how parental education affects the duration of PA and AP of the children and the influence of the place of residence AP of the children.

## Introduction

Physical activity (PA) is defined as any bodily movement produced by the skeletal muscles and resulting in energy expenditure (1) that is greater than at rest (2). It has a positive effect on the physical and mental well-being and the general quality of life (3–6). In addition, the physical and psychological benefits of PA are widely acknowledged (7, 8), while there is not abundant evidence about direct effects of PA on cognition (9). Therefore, researching the influence of PA on the academic performance (AP) is a current research topic of great interest around the world due to increasing evidence about the positive effect of PA on cognitive functioning (10).

The studies on the cognitive benefits focus mainly on the development of learning skills and AP in relation to physical education (PE) and PA (11–13) in children and adolescents (14), which has been developed on the fact that PA increases oxygen saturation (15) and angiogenesis (16, 17) in brain areas responsible for the performance of tasks. Findings from the recent systematic reviews and meta analyses have shown that higher levels of PA are associated with the higher levels of AP (9, 18–20). Not all of these reviews took into account, the methodological quality of the studies included and examined only the AP of the pupils, who were usually assessed by evaluating their knowledge and scholastic aptitude in various subjects, with mathematics and literacy being the most important (21). Recent studies have mostly investigated the impact of classroom breaks and physically active learning (22–24). That is problematic due to different underlying mechanisms of change, such as blood-flow, brain-derived neurotrophic factors, and plasma catecholamines (17, 19, 25–27).

The cognitive and academic training of children is to a large extent a task entrusted to the education system. To improve AP, teaching time for the core academic subjects is extended and protected, often at the expense of time spent in PE and other areas of the curriculum (19). Nevertheless, the fact that Slovenia has one of the best curricula for PE in the world (28, 29), such a trend could potentially also hurt the Slovenian educational system. To our knowledge, PA in Slovenia was usually estimated using questionnaires (30, 31), which are less reliable (32) than objective methods. Only recently have Slovenian researchers begun acquiring such data in a more objective way: with accelerometers (33, 34). Nevertheless, to the best of our knowledge, there is no evidence of a study dealing with the relationship between AP and objectively measured PA.

The aim of the present study was to investigate the relationship between physical fitness (PF), objectively measured PA of Slovenian girls and their AP, which was based on the grade point average (GPA) and separate grades of the four specific subjects.

## Materials and Methods

### Participants

We recruited 20 primary school girls, aged 11–12 years (height 161.3 ± 0.52 cm; weight 52.1 ± 0.9 kg; triceps skinfold 16.3 ± 6.7 mm). Four of them were excluded from the study regarding the rule 70/80 (35) and illness. The study was conducted in accordance with the Declaration of Helsinki and all participants signed informed consent approved by the National Medical Ethics Committee (ID 138/05/13).

### Procedures

#### Physical Fitness

The body height was measured when the subjects were in sportswear and did not wear shoes, using a mechanical stadiometer platform (Seca® 213, Hamburg, Germany) with a small technical error of measurement (TEM = 0.019%). Bodyweight was measured using the electronic scale (Tanita® BC544, Tokyo, Japan; TEM = 0.510%). The skinfold of the triceps was measured halfway between the acromion process and the olecranon process with the Harpenden skinfold caliper.

Physical abilities were determined using the SLOfit test battery. All tests have been evaluated on a sample of Slovenian population and have appropriate dimensional characteristics and are suitable for the use. For every PF indicator, one test was used: sit-ups, standing long jump, sit and reach, running 60 m and 600 m.

#### Physical Activity

In order to objectively assess the level of PA, participants have worn accelerometer Actigraph GT3X (Actigraph LLC, Pensacola, FL) for 5 days, comprising of three school days (Wednesday, Thursday, and Friday) and two weekend days (Saturday and Sunday). Counts signal was sampled in the 15-s epochs in the present study. Data of sleep and awake time were logical and coincided with diary logs and covered school time, extracurricular activities, after-school time and evenings (daytime, excluding mornings, and sleep time). If any accelerometer count was >16,000 counts/min, these were removed, because it is assumed to be beyond biologically plausible range (36). Likewise, if the device recorded the number of counts ≥0, which was constant for 10 min or more, these data were also removed, because it is assumed to be accelerometer malfunction (37). Furthermore, the wear time was calculated regarding the rule 70/80 (38). Wear time was defined from 7:00 to 21:30 (870 min), hence accepted wear time was 609 min or more per day. The sequences of 20 consecutive minutes of zeros were cleaned, because it was considered that ActiGraph was not being worn (39) and the zeros were changed to missing data.

#### Academic Performance

Academic performance was assessed in order to describe the different factors that may influence the success of pupils in school. AP was presented through average grades, calculated from all grades obtained during the whole school year 2014/15 from following school subjects: Mathematics, Slovene language, English language, and PE GPA. GPA was calculated as arithmetic mean grade of average grades from all subjects in that school year.

### Data Collection

The data for the present study were collected at the primary school Ivana Groharja in Škofja Loka. PF data from the school's SLOfit database were evaluated, and PA was measured from Wednesday to Sunday in June 2014 using the ActiGraph GT3X accelerometer. AP was evaluated based on the school documentation provided. In addition, data on additional engagement in sport and the mode of commuting to school were assessed from PA diaries.

### Data Processing and Statistical Analysis

All statistics were made in Microsoft Excel 2007 and IBM SPSS, 20.0. Microsoft Excel 2007 was used for removing the artifacts, counts >16.000, constant values ≥0, and sequences of zeroes, where the sequence was 20 or more zeroes. PA data were processed with the Actilife software (standardized for accelerometer ActiGraph), following the 70/80 rule (38) and non-wear time within a day (40). Testing for distributions of the normality was checked visually (histogram) and with the Kolmogorov–Smirnov test using IBM SPSS Statistics 25.0. Descriptive statistics were calculated for all variables (mainly as mean and standard deviation). Associations between derived estimates of grades, PF, and counts from accelerometer data were computed using the Spearman's correlation. The difference between weekday and weekend PA was assessed using the paired sample *t*-test. The effect size was determined using the Cohen's d. The Chi-square test was used to investigate the correlations between engaging in sport activities and PA and between modes of commuting to school and PA.

## Results

Table 1 shows the evaluated results of the PF of the pupils. It can be seen that the CV% value for the Bent Arm Hang test is very high, which indicates the high dispersion of a frequency distribution.

**Table 1:** Descriptive statistics of the physical fitness of the pupils.

**PF tests**	**Mean**	**SD**	**Min**	**Max**	**CV%**
Taping (n)	42.3	2.8	37.0	47.0	6.7
Long jump (cm)	178.8	27.5	130.0	230.0	15.4
Polygon backwards (s)	13.5	8.7	8.4	45.0	6.5
Sit-ups (n)	49.3	10.8	30.0	63.0	21.9
Stand and reach (cm)	49.0	6.6	35.0	60.0	13.6
Bent arm hang (s)	51.7	36.1	3.0	120.0	69.8
Sprint 60 m (s)	9.9	1.0	8.6	12.2	9.9
Running 600 m (s)	150.8	33.5	109.0	215.0	22.2

*Mean, averaged value; SD, standard deviation; Min, minimum value; Max, maximum value; CV%, coefficient of variation*.

**Figure 1:**
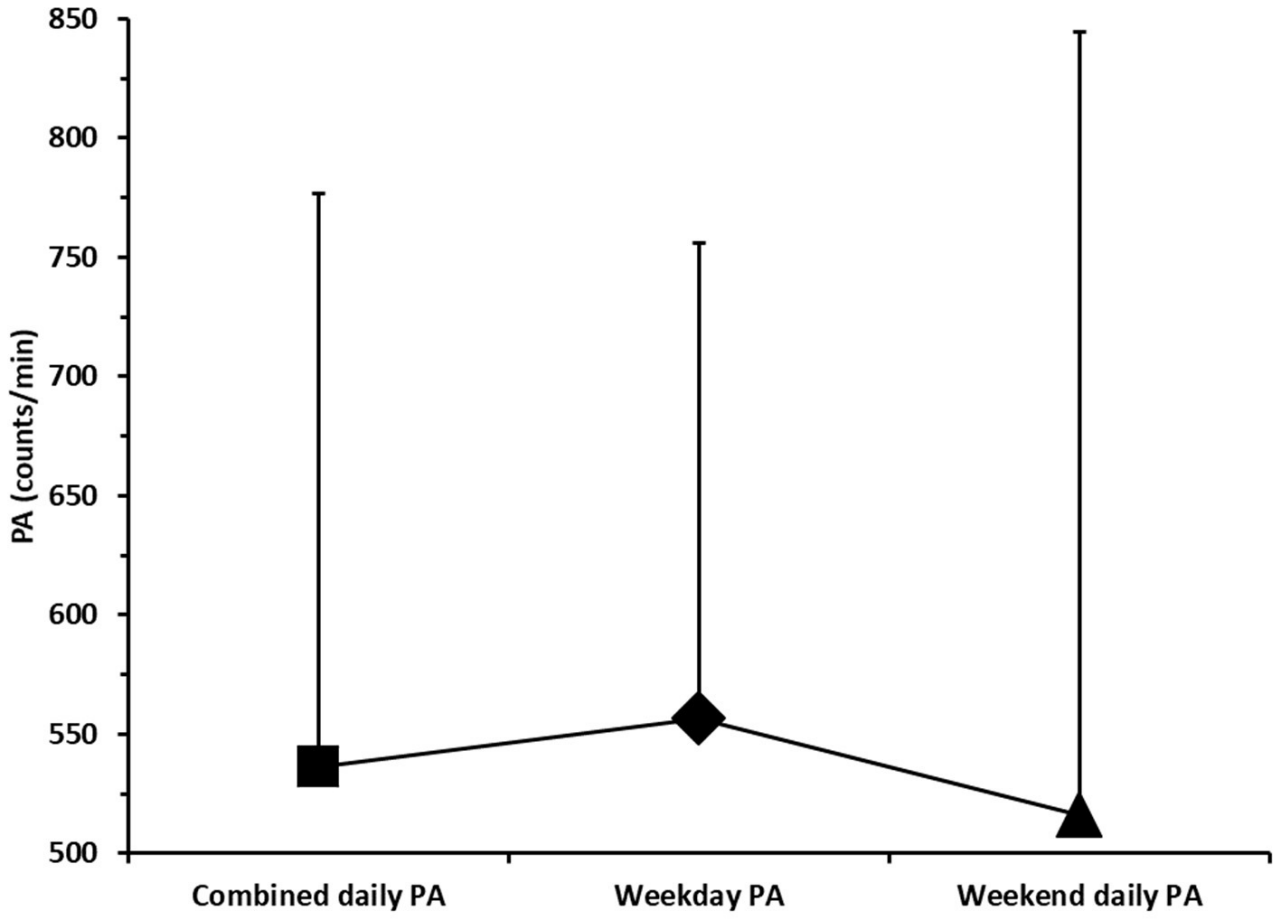
Combined physical activity (PA), weekday PA, and weekend PA of adolescent school girls.

Figure 1 shows that the daily measured PA of pupils reached 536 ± 240 counts/min. The average PA on the weekend days was lower than PA on the school days (516 and 557, respectively). The variance of weekend PA counts was ±328, indicating significant differences in weekend PA among primary school pupils. The standard deviation of PA counts on the weekdays was ±199, indicating a uniform PA pattern of female pupils during the school hours, as the dispersion of the PA counts is lower than on weekends. Regardless of higher PA counts during weekdays, the paired sample *t*-test showed no significant differences in PA counts during weekdays and weekends.

Table 2 shows the prevalence of children taking part in additional sports during the day (in minutes/day) and the way they commute (in minutes/day). The Chi-square test showed no significant correlations between engaging in sport and type of PA and between the modes of commuting and the types of PA (*p* = 0.42 and *p* = 0.12, respectively).

**Table 2:** Sports participation and commuting of included participants.

**PA or sports in min/day**		**Very low PA**	**Low PA**	**Normal PA**	**High PA**
Engaging in sport	Additional sport	12,5%	25,0%	12,5%	50,0%
	Without additional sport	50,0%	12,5%	12,5%	25,0%
Commuting	Active	0,0%	28,6%	14,3%	57,1%
	Driven	55,6%	11,1%	11,1%	22,2%

*Very low PA (<30 min of PA/day); low PA (>30 min PA/day <450 min PA/day); normal PA (>45 min PA/day <60 min PA/day); high PA (>60 min PA/day)*.

As can be seen from the percentage distributions, the children who are engaging in additional sport or PA are reaching recommendations for PA (high PA). Similarly, the children who commuted actively are spending the most time in PA actively. The average grades were calculated for the school year 2014/2015. The calculation of the average grade took into account all grades of an individual school subject in the 2014/15 school year. All grades and descriptive statistics on the AP are shown in Table 3.

**Table 3:** Descriptive statistics for academic performance.

**Subject**	**Mean grade**	**SD**
Mathematics	3.44	0.93
Slovene language	3.78	0.80
English language	3.34	1.02
Second language	3.56	0.85
Physical Education	4.25	0.70
GPA	4.10	0.64

*AP, academic performance; Mean grade, average grade throughout the school year for selected subject or GPA; SD, standard deviation; GPA, grade point average*.

**Figure 2:**
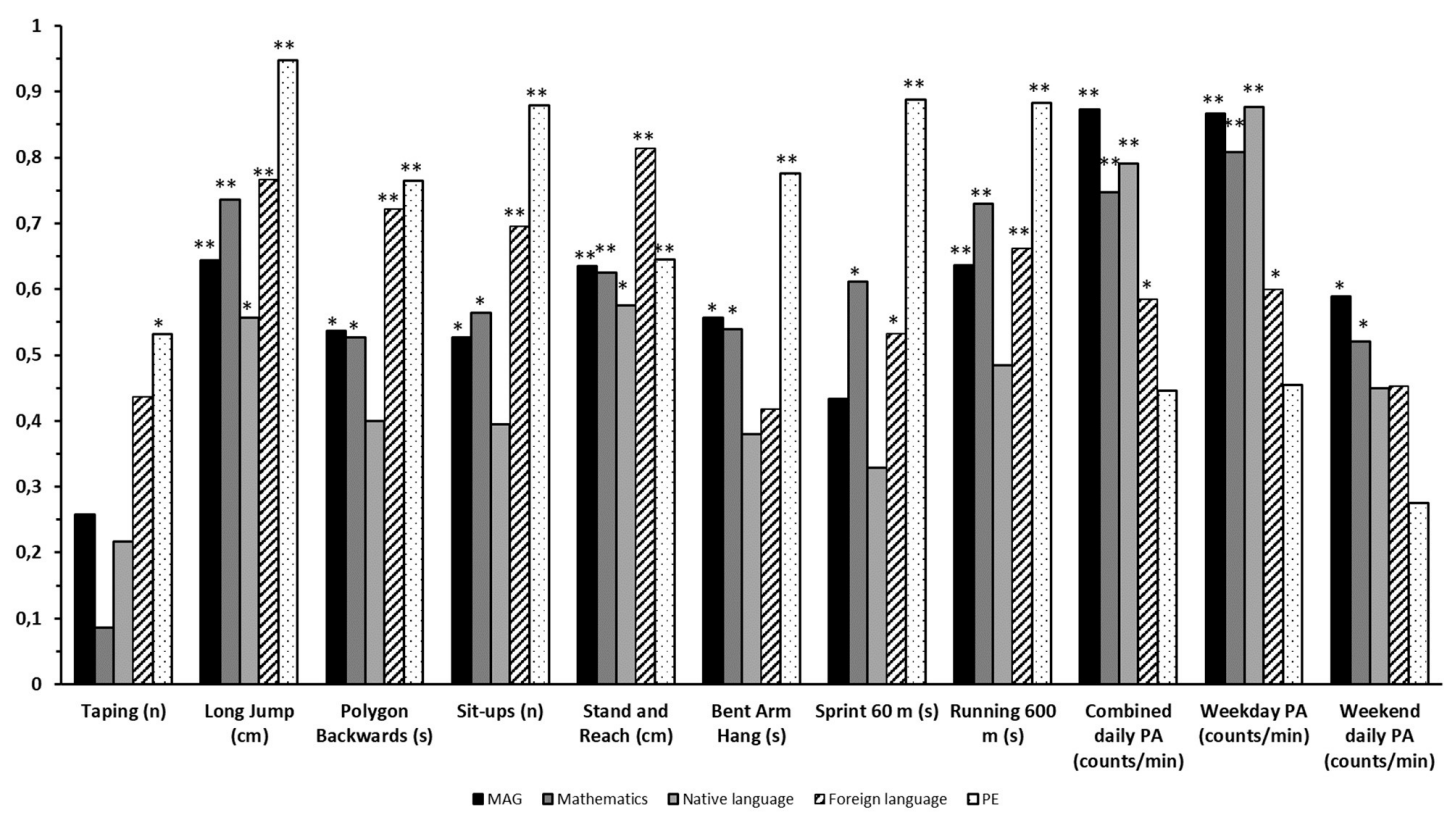
Correlation between physical fitness and academic performance, and physical activity and academic performance. Statistically significant correlations were marked with *p < 0.05 and **p < 0.01.

Correlations between PF and AP and between PA and AP were identified using the Spearman's correlation analysis. Figure 2 shows the above-mentioned correlations for 20 school girls.

Note that the correlation coefficients for the tests Polygon backward, Sprint 60 m and Running 600 m were negative. Nevertheless, we presented them as positive values, since lower values of the above-mentioned tests represented better results and, therefore, correlation was essentially positive. At first glance, it is noticeable that the PE grade shows the highest overall correlations with the results of the PF test battery (*r* = 0.53–0.95) at the significance level 0.01. Nevertheless, correlations surprisingly decrease for the combined daily PA (*r* = 0.45), especially the weekend daily PA (*r* = 0.28). The situation is somewhat different for GPA, where the correlations with PF were mostly moderate (*r* = 0.43–0.64; with the exception of Taping, where *r* = 0.26), while they were moderate to high for PA (*r* = 0.59–0.87). A similar trend was observed for grades in mathematics, native, and foreign languages. The overall lowest correlations with the PF results and one of the highest with PA were found for the grades of the native language.

## Discussion

The results revealed that the girls were more physically active during the weekdays and less active at the weekend (557 vs. 516 counts/min). Despite statistically insignificant differences, the dispersion of results around the average PA during weekdays was lower compared to weekends, indicating more consistent patterns of PA among schoolchildren during the school days. The relatively large dispersion around the average PA could be explained by the relatively small sample size. It can also be explained by the large differences between the minimum and maximum values of the results obtained in a few tests. For example, the worst result in the Bent Arm Hang was only 3 s, which indicates that some of the girls had a large deficit in the upper body strength. In addition, 45 s in Polygon Backward indicates very poor coordination skills.

Compared to many other countries, the Republic of Slovenia has a respectable PE curriculum compared to many other countries (29, 41). PE in the Republic of Slovenia is a standardized and compulsory subject in all primary and secondary schools (41), which is also confirmed when comparing overall activity minutes during weekdays and weekends. According to the results of the present study and following other Slovenian studies (41–43), we can say that the Slovenian primary schoolchildren are physically more active on weekdays, what is also in concordance with other studies (44, 45). In fact, all schools in the Slovenia offer their pupils the opportunity to engage in PA and the majority of Slovenian pupils can be physically active for up to 51 min a day during the school day (41). The decrease at weekends is largely due to a decrease in the intensity of light intensity bouts (44) and the lack of the structured school environment at weekends determine the different PA levels and patterns, which is particularly noticeable among girls (46). As we have included only girls in the present study, the actual PA counts of schoolchildren could be different, probably significantly higher for boys compared to girls, as found in other studies on the Slovenian schoolchildren (42, 47, 48).

Attention-grabbing results were found regarding the correlations between PF, PA, and AP. As expected, the correlation analysis revealed that the pupils with the highest PE grades had the highest level of PF. This could be explained, for example, by knowing that school PE programs routinely allow children to exercise their abdominal muscles and, therefore, a high correlation between PF score and PE grade could be expected. Remarkably, moderate to high correlations were found between the results of PF and GPA, mathematics, native, and foreign languages grades. This is in-line with the previous studies (49, 50).

Surprisingly, however, the same pupils with high grades had a low level of PA. Nevertheless, the trend was the same as for the others—they were more active on weekdays than at weekends. We can only discuss these findings. One reason for this could be that some of the girls took off the accelerometer during the training sessions and games, so this influenced the lower correlation. However, this assumption is probably not true, since the Chi-square test showed that the additional engagement in sports was not a significant factor for the average PA during the day (*p* = 0.12). However, although there was no significant correlation. Table 2 shows that such results could be explained by the mode of commuting to school as an important factor. It has been shown that it is very important to separate commuting from home to school and *vice versa*, as it turned out that children who actively commute from school to home have higher VO2 max (51). A possible explanation could be playing in the playgrounds on the way of home and spending more time in the high-intensity PA. Inactive commuting to school in the morning is often associated with the parental convenience of dropping a child off at school on the way to work, and not necessarily with reservations about the active commuting or an active lifestyle. We speculate that the Chi-square test results would be significant if we had these data separated.

The results should be interpreted with caution and cannot be presented as if the increased PF or PA caused an improved AP or *vice versa*. Many questions arose after the completion of the present study and several new topics opened up, such as the question of how parental education affects the duration of PA and AP of the children and the influence of the place of residence on AP of the children. Self-reported results should be carefully interpreted in terms of the validity and reliability (52) of the measured data, and an objective measurement of PA should be considered to ensure the accuracy of the results. All questions raised in the present study should be considered and analyzed in future studies.

### Strengths and Limitations

The results of this study indicate that there is a significant correlation between AP, PA, and PF for certain outcome measures. However, before outlining the benefits of PA and PF on AP, it is important to note that many factors influence AP. These include socio-economic status, parental involvement, PA, and participation in sport and other demographic factors such as place of residence.

There are methodological limitations to be considered in the current research: (i) the study was conducted with a relatively small sample size (*n* = 20) of schoolchildren; in order to generalize the results to larger groups, the study should have included more participants. Moreover, all the girls were picked from an extracurricular activity called “Healthy lifestyle,” therefore, a potential bias in sample might occur; (ii) the children knew they had been monitored (53), therefore, we did not measure their habitual PA and the results cannot be generalized as a completely realistic measure for PA; (iii) accelerometers are not able to detect the static exercises (54), and the devices might underestimate PA in total; (iv) high device costs and an insufficient number of accelerometers did not allow the measurement of all girls in the same time interval, so that there might be differences in the duration of PA due to different weather conditions; (v) AP was evaluated based on a mathematical grade, Slovenian language grade, a natural science grade, and GPA. AP grades were based on the assessments of the school teachers, therefore, a possible “rater bias” might have been introduced so that the results can only be generalized to those instruments that assess AP; (vi) there was a lack of separate data on the mode of commuting from home to school and *vice versa*.

## Conclusions

The purpose of this study was to investigate the level of PF, objectively measured PA of Slovenian girls, and the relationship between PF, PA, and their AP. The results showed that the girls were more physically active during the weekdays and less active at the weekends. As expected, the correlation analysis showed that the pupils with the highest PE grades had the highest level of PF but had surprisingly low to moderate correlations with the level of PA. The frequency of the PA type in relation to the mode of commuting to school showed that such results could be explained by the mode of commuting to school as an important factor. Based on our previous research, it is assumed that the main factor is commuting from school to home. In addition, moderate to high correlations were found between the results of PF and GPA, mathematics, native, and foreign languages grades. The highest correlation was found between GPA and the combined daily PA. It has been shown that the PE system could influence the future PA and lifestyle of schoolchildren (55) and consequently health status and PF in adulthood.

## Data Availability Statement

The raw data supporting the conclusions of this article will be made available by the authors, without undue reservation.

## Ethics Statement

The studies involving human participants were reviewed and approved by National Medical Ethics Committee (ID 138/05/13). Written informed consent to participate in this study was provided by the participants' legal guardian/next of kin.

## Author Contributions

VS and SĐ conceptualized the study design, recruited subjects into the study, and analyzed and interpreted the data. VZ conducted the research. VS, ŠB, SĐ, and VZ drafted and reviewed the manuscript. All authors have read and approved the final version of the manuscript.

## Funding

This research was funded by the Slovenian Research Agency within the Research project Bio-psycho-social context of kinesiology No. P5-0142 and University Medical Centre tertiary grant TP20210071.

## Conflict of Interest

The authors declare that the research was conducted in the absence of any commercial or financial relationships that could be construed as a potential conflict of interest.

## Publisher's Note

All claims expressed in this article are solely those of the authors and do not necessarily represent those of their affiliated organizations, or those of the publisher, the editors and the reviewers. Any product that may be evaluated in this article, or claim that may be made by its manufacturer, is not guaranteed or endorsed by the publisher.
